# Precision medicine on a budget in Africa: using existing genetic data to mitigate adverse drug reactions to conventional cancer drugs

**DOI:** 10.3389/fbinf.2025.1555637

**Published:** 2025-08-14

**Authors:** Alexandra Lindsey Djomkam Zune, Charles Ochieng’ Olwal, Emmanuel Agbeli, Abdoulaye Baniré Diallo, Emmanuella Amoako, Yaw Bediako, Lily Paemka

**Affiliations:** ^1^ Yemaachi Biotech, Accra, Ghana; ^2^ West African Centre for Cell Biology of Infectious Pathogens (WACCBIP), University of Ghana, Accra, Ghana; ^3^ Department of Biochemistry, Cell and Molecular Biology, College of Basic and Applied Sciences, University of Ghana, Accra, Ghana

**Keywords:** precision medicine, capecitabine, cyclophosphamide, pharmacogenomics, drug metabolizing enzymes

## Abstract

Variations in drug-metabolizing enzymes and transporters are associated with adverse drug reactions (ADRs). ADRs to cancer drugs can differ among populations owing to environmental and genetic differences. Due to limited resources and prohibitive costs associated with drug development, African countries rely on cancer drugs developed from non-African genetic backgrounds. Black Africans carry a high burden of ADRs partly because of the use of poorly optimized drugs. Black Africans are the least studied population despite being the most genetically diverse. There is a profound lack of pharmacogenetic studies in Black African populations, necessitating an urgent need for pharmacogenomic studies in Black African populations to optimize dosing and minimize ADRs. Using two common generic cancer drugs, capecitabine and cyclophosphamide, we leveraged the PharmGKB platform and several genomic databases to highlight the need for pharmacogenomic studies in Africa. Our computational approach identifies previously reported and unreported toxicity- and efficacy-associated variants that are overrepresented or underrepresented in Black Africans relative to other ethnicities. These findings suggest that capecitabine and cyclophosphamide may not work optimally and/or may predispose Black Africans to ADRs. This underscores the need for population-based drug screening and development to minimize ADRs and guarantee better treatment outcomes. Since Black Africans are currently underrepresented in genomic studies, African scientists could adopt our low-cost approach to evaluate the suitability of existing drugs for treating diseases. However, in the long term, African scientists must initiate large-scale genomic studies that will drive the discovery of African-tailored drugs and promote the implementation of precision medicine on the continent.

## Introduction

Drug response is a complex, multifactorial phenomenon that can be attributed to demographic features, environmental factors, clinical backgrounds, and genetic makeup (Taylor et al., 2020). Given that genetics is the major determinant of drug response, the discipline of ‘pharmacogenomics’ emerged to study the genetic determinants influencing drug metabolism, efficacy and adverse drug reactions (ADRs) across individuals. Pharmacogenomics seeks to guide drug selection, dose selection, and/or prioritize patients for closer monitoring through stratification based on genetic variants. This approach could improve drug efficacy and/or safety. The advent of a patient-tailored approach to drug prescription and monitoring, termed ‘precision medicine,’ has improved patient care (Daly, 2017; Micaglio et al., 2021; van der Wouden et al., 2017) but has yet to be adopted in many low-income countries (LICs).

Differences in response to drugs among patients or populations are often attributed to variations in genes encoding drug-metabolizing enzymes. The most common type of genetic polymorphisms in metabolic enzymes is single nucleotide variants (SNVs), which result from variations in the nucleotide base at a single position in the gene encoding the enzyme (Lee, 2010). Variations in drug response are a major challenge in disease management worldwide. It has been estimated that 25%–50% of patients have an unfavorable response to various drugs (Spear et al., 2001). These variations may result in reduced drug efficacy and/or ADRs. Nearly 15% of all hospital admissions are associated with one or more ADRs (Bouvy et al., 2015; Giardina et al., 2018). Studies have indicated that certain races or ethnicities are more likely to experience ADRs compared to others. For instance, a systematic review reported that Asians, Black Africans and Caucasians are predisposed to anticoagulant-related adverse drug events (ADEs), diabetes agents-related ADEs and opioid-related ADEs, respectively (Baehr et al., 2015). Another study reported that Black Africans and Hispanic have a higher number of platin-associated pharmacogenomic variants associated with toxicity than Caucasians (Jordan et al., 2022). A systematic review by McDowell et al. (2006) reported that Black Africans are three times more likely to experience angioedema compared to non-Blacks when using angiotensin-converting enzyme inhibitors. McDowell and colleagues further noted a doubled risk of intracranial bleeding in Black Africans compared to non-Black people during thrombolytic therapy. The aforementioned reports highlight disparities among races/ethnicities, emphasizing the need for a deeper understanding of the factors underlying varied responses to medications.

Owing to the prohibitive costs and technology associated with drug development, many LICs rely on medications from high-income countries (HICs). However, due to inherent genetic differences among ethnicities, such drugs may be less efficacious or pose serious ADRs when administered to individuals in LICs where the drugs have not been optimized. This view is supported by previous reports, which demonstrated that warfarin dosage responses are largely influenced by ethnicity. For instance, the average daily warfarin doses differ, with individuals of African ancestry requiring the highest daily dose (5.7 mg) to achieve a stable therapeutic international normalized ratio, compared to 5.1 mg in Europeans, 4.4 mg in Latinos, and 3.4 mg in individuals of Asian ancestry (Kaye et al., 2017). Despite the existing evidence on the differences in drug responses across populations, pharmacogenomics remains in its infancy in LICs. This has contributed to severe drug reactions and deaths due to the administration of inappropriate drugs and/or dosage (Mosher et al., 2010; Pinheiro et al., 2016; Schneider et al., 2017; Wright et al., 2014). Among the 300-plus medications with FDA-labeled pharmacogenetic product information and the 100 small molecule-medications with clinical pharmacogenetics guidelines, a review by Radouani et al. (2020) reported that only a few have been studied in African populations. Moreover, despite the continent’s vastness and a population of over 1 billion people, encompassing a wide range of ethnically diverse populations, these drugs have only been investigated in 1–3 studies on average. This highlights a significant gap in knowledge and a pressing need to study drug metabolism and disposition in African populations to optimize dosing and minimize ADRs. This need becomes even more apparent in reviews that emphasize the differences in drug metabolizing enzymes across ethnicities. For example, Rajman et al. (2017) identified CYP alleles (CYP2B6*6, CYP2C8*2, CYP2D6*3, CYP2D6*17, CYP2D6*29, CYP3A5*6, and CYP3A5*7) of potential clinical relevance displaying a marked difference in distribution in Black Africans compared to Asian or Caucasian populations. Consequently, pharmacogenomic screening of Black African patients is crucial to determine their suitability for certain drugs.

The Clinical Pharmacogenetics Implementation Consortium (CPIC) has published more than 100 Level A gene–drug guidelines (Abdullah-Koolmees et al., 2021), with only a few addressing cancer drugs such as capecitabine, a *DPYD*-guided drug. However, in African settings, capecitabine is administered without genetic testing due to uncharacterized allele frequencies and limited infrastructure (Radouani et al., 2020). Cyclophosphamide, on the other hand, is a provisional Level D drug (i.e., no prescribing action recommended). Due to the low cost of cyclophosphamide and capecitabine, they are widely administered in sub-Saharan Africa to breast, colorectal and lymphoid cancer patients (Baissa et al., 2025; Ahmed et al., 2020; Herbst et al., 2018). Although some population-level differences in ADRs to capecitabine have been documented (Brazelton et al., 2022), the drugs are being used without substantial data on the likelihood of ADRs among Black Africans. To highlight the need for pharmacogenetic testing of drugs, particularly cancer drugs in genomically understudied populations, we compared the allele frequencies of variants associated with capecitabine and cyclophosphamide toxicity and efficacy across four populations. Our low-cost computational analysis uncovered some variants associated with toxicity or efficacy that are under- or over-represented in Black Africans relative to other ethnicities. These findings underscore the need for pharmacogenomic studies on both new and old drugs among Africans to guarantee their efficacy and limit ADRs. Furthermore, this innovative approach could also be extended to other drugs, broadening its applicability and impact.

## Methods

### Data acquisition

Data for this study were primarily mined from the PharmGKB platform (https://www.pharmgkb.org/), which curates pharmacogenetic data for clinicians and researchers. Using an automated Python script, we first pulled out SNVs associated with capecitabine (Supplementary Table S1) and cyclophosphamide (Supplementary Table S2) toxicity and efficacy. We then downloaded the allele frequencies of the SNVs from major multi-ethnic genomic studies on The National Center for Biotechnology Information (NCBI) as seen in Supplementary Tables S3 and S4). The CSV files downloaded were subsequently loaded into the R software for visualization. In brief, we filtered the PharmGKB datasets (Supplementary Tables S1 and S2) to obtain variants associated with capecitabine and cyclophosphamide toxicity or efficacy. We then annotated the allele frequencies of the toxicity- and efficacy-associated variants from six multi-ethnic studies (i.e., ExAC, gnomAD-Exomes, gnomAD-Genomes, 1,000 Genomes, HapMap, The PAGE Study) from NCBI (Supplementary Tables S3 and S4). Finally, the allele frequencies of the variants across four major ethnicities (i.e., Black Africans, Asians, Caucasians and Hispanics) were visualized as heatmaps and compared statistically. For capecitabine, 166 unique SNVs (i.e., 103 toxicity-associated; 63 efficacy-associated) were extracted. After filtering across the six studies, we identified 160 SNVs: 100 toxicity-associated and 60 efficacy-associated SNVs. All the 160 SNVs had allele frequency data for Black Africans. For cyclophosphamide, 189 unique SNVs (i.e., 147 for toxicity and 42 for efficacy) were retrieved; 180 SNVs remained after filtering across the six studies: 139 and 41 toxicity-associated and efficacy-associated SNVs, respectively. All the 180 SNVs had allele frequency data for Black Africans. The sample sizes of the populations across the six genomic studies are summarized in Table 1.

**TABLE 1:** Population sample sizes across genomic studies.

Study	Black African	Asian	Caucasian	Hispanic
Capecitabine				
ExAC	9,728	23,916	10,784	NP
gnomAD-Exomes	15,903	48,488	59,009	NP
gnomAD-Genomes	41,615	3,111	30,676	NP
1000 Genomes	1,322	1986	694	NP
HapMap	511	209	591	NP
The PAGE	NP	4,584	2,895	5,200
Cyclophosphamide				
ExAC	9,373	23,019	10,472	NP
gnomAD-Exomes	15,233	46,142	56,633	NP
gnomAD-Genomes	41,861	3,122	30,893	NP
1000 Genomes	1,322	993	694	NP
HapMap	536	205	608	NP
The PAGE	NP	4,585	2,896	5,200

Note: The values represent the average sample size from which variant allele frequencies were obtained.

NP, not provided.

### Data analysis, visualization and code availability

Bioinformatics analyses were performed primarily using ggplot2 v3.5.2, ComplexHeatmap v2.25.0 packages anchored in R version 4.5.0 (R Development Core Team, Vienna, Austria) and RStudio v2025.5.0.496. Using the Wilcoxon test from the ggsignif package v0.6.4, we statistically compared the allele frequencies of Black Africans versus Asians, Caucasians or Hispanics. A *p*-value <0.05 was considered significant. Python codes used to extract datasets for the study are available at https://gitfront.io/r/Agbeli/3XCD8Hd3zMGr/precision-medicine/. No custom R codes were used in this study.

## Results

### Several SNVs associated with capecitabine and cyclophosphamide toxicity are overrepresented in Black Africans

**FIGURE 1:**
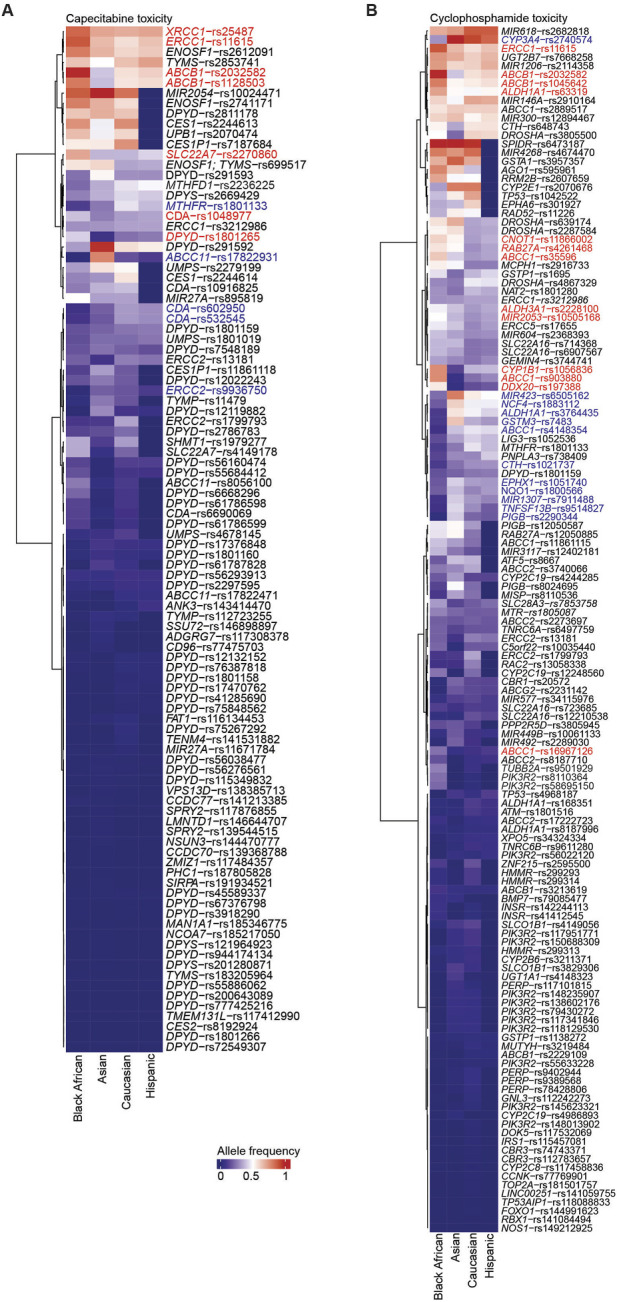
SNVs associated with capecitabine and cyclophosphamide toxicity across ethnicities. Heatmap summarizing the allele frequencies of SNVs associated with capecitabine **(A)** and cyclophosphamide **(B)** toxicity across four ethnicities. The values on the heatmap are the median allele frequencies for six genomic studies (i.e., ExAC, gnomAD-Exomes, gnomAD-Genomes, 1,000 Genomes, HapMap, The PAGE Study). The SNVs overrepresented and underrepresented are highlighted in red and blue, respectively. Only variants with allele frequencies across the four populations are highlighted.

**FIGURE 2:**
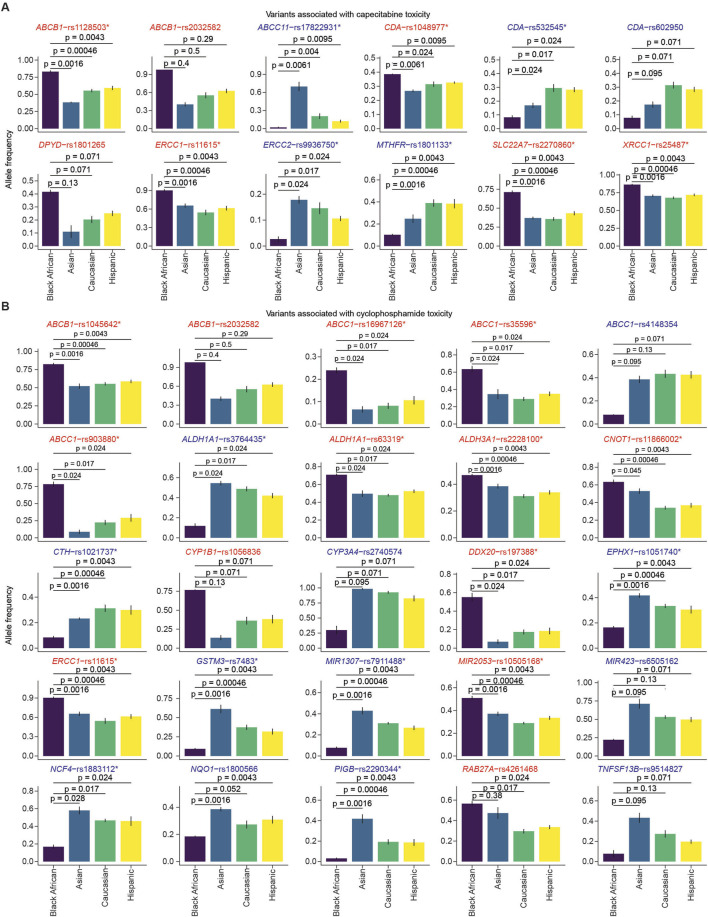
Comparison of allele frequencies of variants associated with capecitabine and cyclophosphamide toxicity across ethnicities. The bar plots show the minor‐allele frequencies of SNVs associated with capecitabine **(A)** and cyclophosphamide **(B)** toxicity across four ethnic groups (Black African, Asian, Caucasian, Hispanic). Statistical comparison between Black Africans versus each of the three ethnicities was performed using the Wilcoxon test. SNVs that had significantly higher or lower allele frequencies compared to all the other ethnicities are marked with asterisks. The bars represent the mean allele frequencies from the six studies (i.e., ExAC, gnomAD-Exomes, gnomAD-Genomes, 1,000 Genomes, HapMap, The PAGE Study), with error bars indicating the standard error. The SNVs overrepresented and underrepresented are highlighted in red and blue, respectively.

Our initial assessment focused on identifying SNVs associated with capecitabine or cyclophosphamide toxicity in Black Africans, and to compare these findings with other ethnicities. To achieve this, we retrieved variants associated with the toxicity and efficacy of the two drugs curated in PharmGKB (Supplementary Tables S1 and S2; https://www.pharmgkb.org/). From the PharmGKB database, we pulled datasets providing the allele frequencies of variants across ethnicities obtained from major studies including gnomAD, 1000Genomes and ExAC (Supplementary Tables S3 and S4). Our analyses identified several SNVs associated with capecitabine toxicity that are either overrepresented or underrepresented in Black Africans relative to Asians, Caucasians or Hispanics (Figure 1A). Statistical comparisons uncovered certain variants (i.e., *XRCC1*-rs25487, *ERCC1*-rs11615, *ABCB1*-rs1128503, *SLC22A7*-rs2270860, *CDA*-rs1048977, *DPYD*-rs1801265) associated with capecitabine toxicity that were significantly overrepresented in Black Africans compared to the other three ethnicities (Figure 2A). Concerning cyclophosphamide, our analysis also identified several SNVs associated with toxicity that were either overrepresented or underrepresented in Black Africans relative to Asians, Caucasians or Hispanics (Figure 1B). A statistical comparison of the allele frequencies of the variants across ethnicities showed that 10 variants (i.e., *ALDH1A1*-rs3764435, *RAB27A*-rs4261468, *ABCC1*-rs35596, *CYP1B1*-rs1056836, *ABCC1*-rs903880, *DDX20*-rs197388, *ERCC1*-rs11615, *ABCB1*-rs2032582, *ABCB1*-rs1045642, *ALDH1A1*-rs63319) associated with toxicity were significantly overrepresented in Black Africans compared to the other ethnicities (Figure 2B). Taken together, these findings suggest that Black Africans have a higher predisposition to adverse events associated with capecitabine and/or cyclophosphamide compared to other ethnicities.

### Variants associated with capecitabine and cyclophosphamide efficacy are underrepresented in Black Africans

**FIGURE 3:**
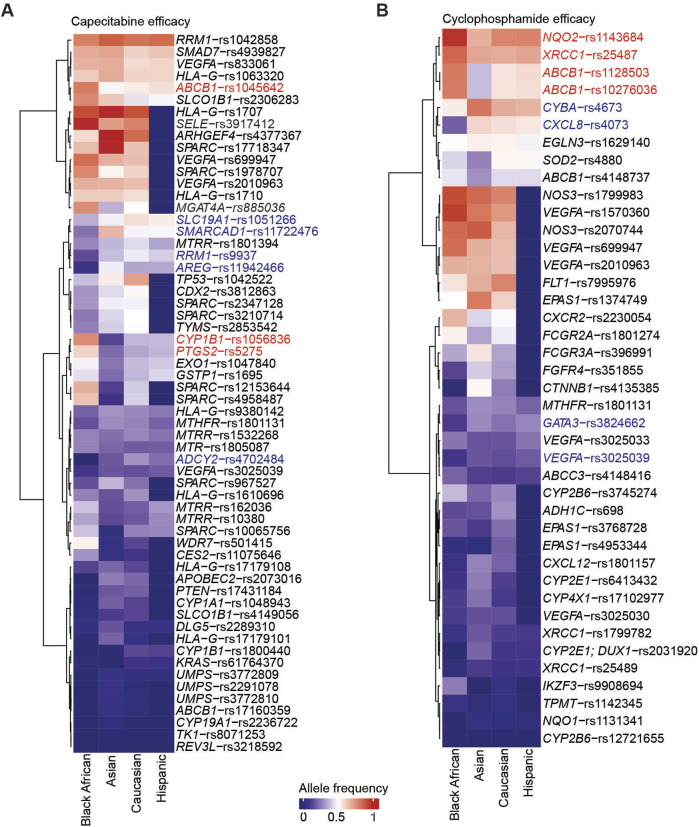
SNVs associated with capecitabine and cyclophosphamide efficacy across ethnicities. Heatmap summarizing the allele frequencies of SNVs associated with capecitabine **(A)** and cyclophosphamide **(B)** efficacy across four ethnicities. The values on the heatmap are the median allele frequencies for six genomic studies. The SNVs overrepresented and underrepresented are highlighted in red and blue, respectively. Only variants with allele frequencies across the four populations are highlighted.

**FIGURE 4:**
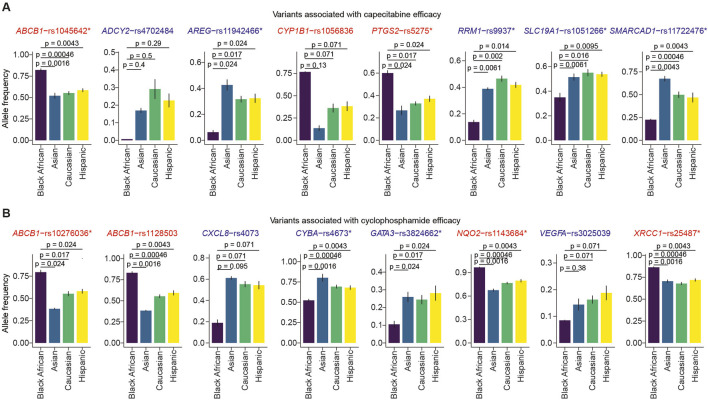
Comparison of allele frequencies of variants associated with capecitabine and cyclophosphamide efficacy across ethnicities. The bar plots show the minor‐allele frequencies of SNVs associated with capecitabine **(A)** and cyclophosphamide **(B)** efficacy across four ethnic groups (Black African, Asian, Caucasian, Hispanic). Statistical comparison between Black Africans versus each of the three ethnicities was performed using the Wilcoxon test. SNVs with significantly higher or lower allele frequencies compared to the other ethnicities are marked with asterisks. The bars represent the mean allele frequencies from six genomic studies, while error bars depict the standard error. The SNVs overrepresented and underrepresented are highlighted in red and blue, respectively.

We next sought to identify SNVs associated with capecitabine or cyclophosphamide efficacy in Black Africans, comparing them to other ethnicities using the same analysis described for toxicity. We identified SNVs associated with capecitabine efficacy that are either overrepresented or underrepresented in Black Africans compared to Asians, Caucasians or Hispanics (Figure 3A). Additionally, statistical comparisons identified four variants (i.e., *SLC19A1*-rs1051266, *SMARCAD1*-rs11722476, *RRM1*-rs9937, *AREG*-rs11942466) associated with capecitabine efficacy that were significantly underrepresented in Black Africans compared to Asians, Caucasians or Hispanics (Figure 4A).

Repeating the analysis for cyclophosphamide, we identified SNVs associated with toxicity that were overrepresented or underrepresented in Black Africans relative to Asians, Caucasians or Hispanics (Figure 3B). Statistical comparisons of variant allele frequencies across ethnicities revealed that *GATA3*-rs3824662 and *VEGFA*-rs3025039 were significantly underrepresented in Black Africans compared to the other three ethnicities (Figure 4B). Taken together, these findings suggest that capecitabine and/or cyclophosphamide may be less efficacious in Black Africans as certain variants associated with efficacy seem to be underrepresented in this population.

## Discussion

Due to Africa’s high genetic diversity and overdependence on drugs from developed countries, individuals of African ancestry are more susceptible to ADR-related challenges. In this brief research report, we present several variants associated with the toxicity or efficacy of two cancer drugs (i.e., capecitabine and cyclophosphamide) commonly administered in African countries (Kizub et al., 2022).

The present study demonstrated that allele frequencies of SNVs associated with capecitabine or cyclophosphamide toxicity vary across ethnicities, predicting differences in ADRs among patients from these ethnic backgrounds. Notably, our bioinformatics analysis indicated that *XRCC1*-rs25487, *ERCC1*-rs11615, *ABCB1*-rs1128503, *SLC22A7*-rs2270860, *CDA*-rs1048977 and *DPYD*-rs1801265, which confer toxicity to capecitabine, are overrepresented in Black Africans. Similarly, we found *ALDH1A1*-rs3764435, *RAB27A*-rs4261468, *ABCC1*-rs35596, *CYP1B1*-rs1056836, *ABCC1*-rs903880, *DDX20*-rs197388, *ERCC1*-rs11615, *ABCB1*-rs2032582, *ABCB1*-rs1045642 and *ALDH1A1*-rs63319, which are known to confer toxicity to cyclophosphamide to be overrepresented in Black Africans. A previous study conducted in Spain confirmed the link between some of these SNVs (i.e., *CDA* rs1048977 and *SLC22A7* rs2270860) with severe capecitabine toxicity (Pellicer et al., 2017). Another study conducted in Spain also linked *ABCB1*-rs1128503, rs2032582, and rs1045642 with a high risk of grade >2 diarrhea and overall toxicity upon capecitabine administration for colorectal cancer (Garc et al., 2015). A North American study reported that *ABCC1*-rs903880, *ALDH1A1*-rs3764435 and *ALDH1A1*-rs63319 are associated with severe hematological toxicity after administration of doxorubicin and cyclophosphamide (Yao et al., 2014). Regarding efficacy, several SNVs associated with efficacy to capecitabine (i.e., *SLC19A1*-rs1051266, *SMARCAD1*-rs11722476, *RRM1*-rs9937, *AREG*-rs11942466) and cyclophosphamide (i.e., *GATA3*-rs3824662, *VEGFA*-rs3025039) were underrepresented in Black Africans compared to other ethnicities. The present findings are consistent with previous studies conducted on other drugs. For example, a study associated *SLC19A1*-rs1051266 with methotrexate efficacy (Eektimmerman et al., 2020). Notably, many of the SNVs identified in our study have not been reported in pharmacogenetic studies. Further investigation is needed to elucidate the impact of these unknown variants on toxicity or efficacy. The SNVs highlighted in this study could serve as potential pharmacogenomic markers for increased ADRs or reduced efficacy.

In the present study, we identified several toxicity-associated (Figures 1, 2) and efficacy-associated (Figures 3, 4) SNVs that are underrepresented and overrepresented in Black Africans. These findings indicate that Black Africans carry certain variants that may reduce toxicity or increase efficacy to capecitabine and/or cyclophosphamide, suggesting a differential response to these drugs among Black Africans. Thus, the blanket administration of these drugs in this population could have serious ramifications. Taken together, our findings underscore the need for pharmacogenetic testing of these variants in Black African patients prior to the administration of specific drugs to avoid severe ADRs and ensure optimal therapeutic benefit.

Due to the substantial resources and extended duration required to conduct pharmacogenomic studies and generate African-tailored drugs, the African continent will continue to depend on drugs developed from genomic studies conducted in other populations. However, as demonstrated in this study, the dependence on such drugs is likely to pose serious health challenges since Black Africans are more genetically diverse and are more likely to carry more variants, including those that predispose them to ADRs. This calls for policymakers and researchers in these genomically underrepresented settings to evaluate the suitability of cancer drugs from the HICs. In this study, we propose an approach for identifying variants that may confer toxicity or efficacy to a drug of interest before its administration to a patient. Our approach could be utilized to identify candidate variants for inclusion in pharmacogenetic screening in low-resource settings. This will ensure that efficacious and less toxic drugs are administered, resulting in better cancer management and reduced ADRs.

In this study, we aimed to highlight the need for pharmacogenetic testing of cancer drugs in genomically understudied populations, such as Africa, by identifying candidate variants unique to Africans for inclusion in pharmacogenetic screening. However, we recognize that this approach may not have an immediate impact on cancer management. Our analysis identified several toxicity- and efficacy-associated variants that had similar allele frequencies across the four populations, indicating an opportunity to utilize existing pharmacogenetic data. Since drug metabolism or pharmacokinetics involve conserved molecular pathways across humans, Africans could benefit from the similarities in allele frequencies of certain variants and adopt pharmacogenetic screening protocols based on guidelines established in HICs. For example, cancer drugs with actionable drug-gene pairs (*e.g*., *CYP2D6*-tamoxifen, *DPYD*-fluoroucil, *DPYD*-tegafur, *UGT1A1*-irinotecan, *DPYD*-capecitabine) could be considered. Rather than focusing on the differential allele frequencies in variants of these genes, Africa could target variants with similar allele frequencies across populations for pharmacogenetic screening before administering drugs like tamoxifen, tegafur, capecitabine, among others. This approach could provide immediate benefits for Africans as they await large-scale genomic studies that will inform the development of African-tailored therapies.

In summary, we performed computational analysis using multi-ethnic genomic data integrated with variants associated with capecitabine and cyclophosphamide toxicity and efficacy. Our analysis identified variants with differing allele frequencies in Black Africans compared to other populations, some of which may predispose Black Africans to capecitabine and cyclophosphamide-associated ADRs. Additionally, we identified several variants with similar allele frequencies across the four populations. Variants with unique allele frequencies in Africans could be prioritized for pharmacogenetic testing in this population. For immediate impact and to address the growing burden of cancers, African countries could also focus on pharmacogenetic screening of variants with similar allele frequencies, leveraging well-established guidelines from HICs. Both strategies will ensure that African cancer patients receive less toxic and more efficacious drugs. Even though our conclusions may be limited by the imbalanced sample sizes across populations, our study provides a low-budget approach through which many of the drugs developed in non-Africans could be screened for use, not only in Black Africans but also in other LICs, to identify possible markers for drug toxicity and efficacy. However, in the long term, it is imperative to conduct large-scale pharmacogenomic studies among Black populations to address the existing knowledge gap and pave the way for African-tailored cancer therapies and drive the advancement of personalized treatments for cancer and other terminal diseases.

## Data Availability

The original contributions presented in the study are included in the article/Supplementary Material, further inquiries can be directed to the corresponding authors.
